# What is your diagnosis?

**DOI:** 10.4274/jtgga.galenos.2020.2019.0184

**Published:** 2020-09-03

**Authors:** Latika Chawla, Aditi Jindal, Prashant Kumar Verma, Anupama Bahadur, Shashi Prateek

**Affiliations:** 1 Department of Obstetrics and Gynecology, All India Institute of Medical Sciences, Rishikesh, India; 2 Department of Pediatrics, Division of Genetics, All India Institute of Medical Sciences, Rishikesh, India

A 33 year-old, gravida three with two previous living issues was referred to our institute at 31 weeks period of gestation (POG) with an ultrasound finding of polyhydramnios. The patient lived in a remote hilly area of the state and she did not seek any antenatal care in the first or second trimesters. On account of polyhydramnios, we performed a detailed anatomical survey of the fetus by 2-dimensional (2D) ultrasonography, and we were confronted by the following subtle abnormalities: brachycephaly (Figure 1) with borderline increased cephalic index (85.3%); prominent and easy visualization of the eye balls and eyelids suggestive of orbital proptosis (Figure 2, 3); mildly depressed nasal bridge with a beaked nose (Figure 4). Binocular and interocular distances were normal. All four limbs were normal. No other structural defect could be identified in the fetus. Maximum vertical amniotic fluid pocket was only 6 cm (1).

The husband reached the hospital only a day later. Interestingly the father also exhibited similar dysmorphic facial features. The father reported that the couple’s two older children, a boy and a girl, looked like him too. He had a broad head with exophthalmos, widely separated and deviated eye balls, beaked nose and mid-facial hypoplasia. His intelligence was normal and there was no associated structural defect in any other body part.

## Answer

**Figure 5:**
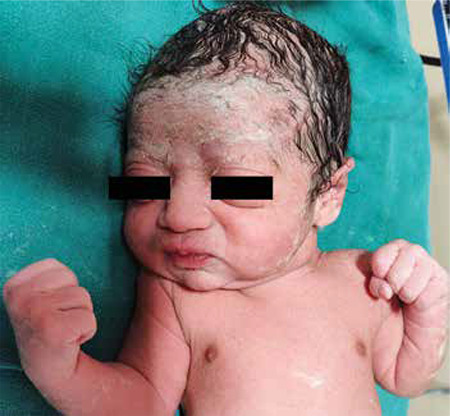
Subtle dysmorphic features of the neonate including a broad head, orbital proptosis, depressed nasal bridge, and beaked nose consistent with typical phenotype of Crouzon syndrome

The patient underwent a spontaneous normal vaginal delivery at 38 weeks and delivered a male baby of 3.1 kilograms. Baby (Figure 5) was born with all previously mentioned subtle dysmorphic features. Baby and father were evaluated by the geneticist at the institute. The phenotypic features of father, two previous children, this baby and the suggestive inheritance pattern all pointed towards a diagnosis of Crouzon syndrome. The family did not opt for genetic analysis due to financial constraints.

Crouzon syndrome is a rare disorder with an incidence of 15-16 cases per million live births (2). It is an autosomal dominant disorder, although sporadic cases have also been reported. It occurs due to mutations in the* fibroblast growth factor receptor 2 *gene located on chromosome 10.

Facial dysmorphism and related abnormalities in craniosynostosis depend upon which and how many of the cranial sutures fuse prematurely. Even after premature fusion of the sutures, which may occur in utero or after birth, the brain continues to grow along the plane of the remaining open sutures and the pressure gives an abnormal appearance and shape to the skull (3,4,5). Crouzon syndrome is most commonly associated with bi-coronal synostosis that gives the typical brachycephaly. Other cranial sutures may fuse as well. Abnormal premature fusion of various sutures of the base of the skull and face is associated with mid-facial hypoplasia and beaked nose (psitticorhinia). Retrusion of lateral and inferior orbital margins results in shallow orbits leading to proptosis and exotropia (6). Structural abnormalities of the ear, such as narrowing and stenosis of the ear canal, may lead to hearing loss. Arnold Chiari malformation is reported to be common in patients of Crouzon syndrome (3).

Affected babies should be followed up for development of ventriculomegaly and increased intracranial pressure. Choanal atresia and abnormalities of the upper airway may lead to life threatening respiratory distress at birth. Since it is an autosomal dominant disorder with variable expression, the phenotype may be variable, even in members of the same family, with certain members having more prominent features and others having subtle dysmorphism, as was in our case. The baby born in our case had most of the typical characteristic features (brachycephaly, proptosis, exophoria and beaked nose) however there was no hypertelorism or mid-facial hypoplasia. Even the exophthalmos was very subtle.

Prenatal diagnosis of cranio-synostotic syndromes has been deemed difficult, especially when gross abnormalities of fetal head are not present (7). Even though Crouzon syndrome is the most common of the cranio-synostotic syndromes, its prenatal diagnosis is extremely challenging and has rarely been reported previously, as the skull abnormalities may be very mild and there is lack of associated limb abnormalities (7). To the best of our knowledge there are only 6 reports of prenatal diagnosis of Crouzon syndrome by ultrasonography in the current literature. Prenatal diagnosis of Crouzon syndrome was first made in 1989 by Menashe et al. (8) in a 35 weeks POG fetus where exophthalmos was the only facial abnormality detected on ultrasound. Leo et al. (9) diagnosed Crouzon syndrome in a 16 weeks POG fetus with increased binocular and interocular diameters. In 1993, Gollin et al. (10) identified a fetus with Crouzon syndrome at 23 weeks with cloverleaf skull, exophthalmos, hypertelorism with mild ventriculomegaly. The same year, Escobar et al. (11) reported prenatal diagnosis of another fetus with Crouzon syndrome at 20 weeks POG and with a positive family history. In 2002, Miller et al. (12) published a retrospective study where they reported prenatal diagnosis of two cases, at 20 and 22 weeks by noting brachycephaly and hypertelorism. Nørgaard et al. (13) in 2011 reported prenatal diagnosis of Crouzon syndrome in a 35-week fetus using both 2D and 3D ultrasonography.

It is important to identify cranio-synostotic syndromes prenatally for the following reasons. Firstly, if diagnosis made is within the legal limit, parents may be offered an option of a medical termination of pregnancy. Secondly, parents should be prepared for the birth of the child with special needs. Pre-natal identification of such syndromes will facilitate in utero transfer/referral of the mother to a tertiary care center equipped with facilities for multidisciplinary management of such syndromic babies, including neonatology, geneticist, neurosurgeon, and oro-maxillofacial surgeons. Finally, with prior notice a team can be ready for immediate management of possible respiratory compromise at birth in such affected babies.

## Figures and Tables

**Figure 1:**
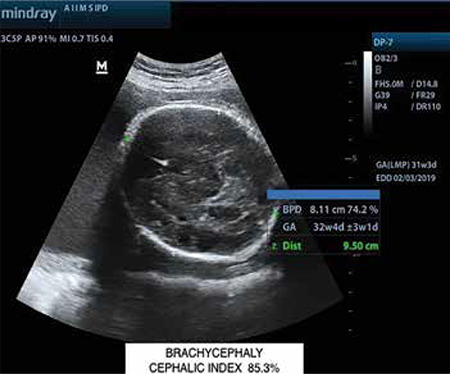
Ultrasound image (2-dimensional) of fetal head showing brachycephaly with cephalic index more than 85% [Biparietal diameter (BPD): 8.11 cm, Occipito-frontal diameter (OFD): 9.5 cm, Cephalic index (BPD/OFD) x100: 85.4%] BPD: Biparietal diameter

**Figure 2:**
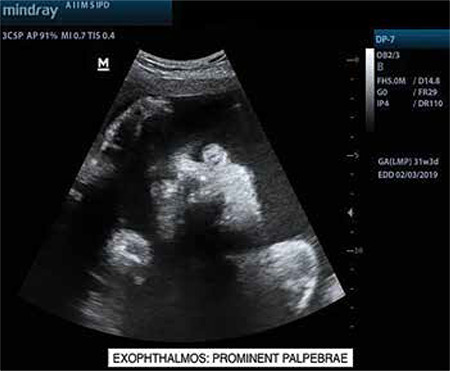
Ultrasound image (2-dimensional) of coronal view of fetal face demonstrating easily visible eyeballs and palpebrae, suggestive of orbital proptosis

**Figure 3:**
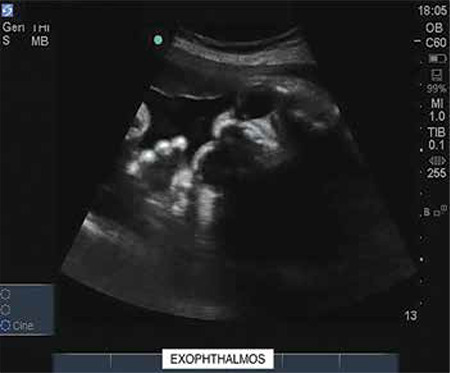
Ultrasound image (2-dimensional) of transverse section of fetal face demonstrating bulging eyeballs, suggestive of orbital proptosis

**Figure 4:**
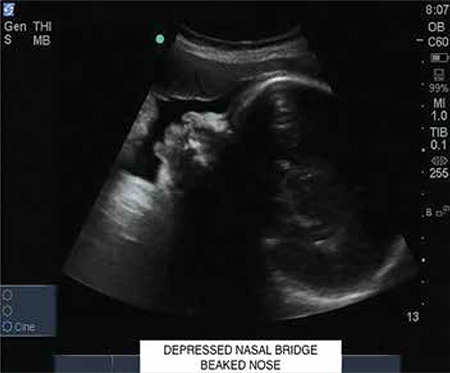
Ultrasound image (2-dimensional) of fetal facial profile showing mildly depressed nasal bridge with a beaked nose
